# Lipopolysaccharide clustering in colistin persistent and resistant bacteria

**DOI:** 10.1038/s44259-025-00158-4

**Published:** 2025-10-27

**Authors:** Fengyi Wang, George Merces, Dominic Alderson, Adam J. M. Wollman, Mark Geoghegan, Chien-Yi Chang

**Affiliations:** 1 School of Dental Sciences, Faculty of Medical Sciences, Newcastle University, Newcastle Upon Tyne, UK; 2 Biosciences Institute, Faculty of Medical Sciences, Newcastle University, Newcastle Upon Tyne, UK; 3 Image Analysis Unit, Faculty of Medical Sciences, Newcastle University, Newcastle Upon Tyne, UK; 4 School of Engineering, Newcastle University, Newcastle Upon Tyne, UK

**Keywords:** Antimicrobials, Bacteria

## Abstract

Polymyxin antibiotics target lipopolysaccharides (LPS) in Gram-negative bacteria, but persistence and *mcr*-mediated resistance increasingly compromise their therapeutic efficacy. Here, we use super-resolution localisation microscopy to investigate the nanoscale organisation of LPS and membrane lipids in colistin-persistent *Escherichia coli* and *Pseudomonas aeruginosa*. We find that persister cells exhibit heterogeneous LPS and membrane lipid distributions, with localised LPS clustering along the cell envelope, compared to susceptible cells. Unexpectedly, *mcr-1*-positive *E. coli* displays a similar LPS clustering phenotype to that of persisters, without altering membrane organisation. These findings suggest that both persistence and resistance involve envelope reorganisation, with increased LPS clustering and remodelling observed in both *E. coli* and *P. aeruginosa*. This work reveals a morphological signature of persistence and identifies a shared feature between persistence and resistance in Gram-negative bacteria.

## Introduction

Polymyxins, including colistin and polymyxin-B, are clinically used, with colistin serving as a last-resort treatment for multidrug-resistant Gram-negative bacteria. Their primary target is lipopolysaccharide (LPS), a major structural component of the outer leaflet of the Gram-negative outer membrane (OM), composed of an O-antigen, a core polysaccharide, and lipid A^1,2^. Lipid A carries negatively charged phosphate groups that are stabilised by divalent cations (Ca^2+^ and Mg^2+^)^3^. Colistin, a cyclic lipopeptide consisting of a fatty acyl chain, a linear tripeptide, and a cyclic heptapeptide enriched in diamino butyric acid (DAB), displaces these cations via its positively charged DAB residues and binds electrostatically to lipid A^4^. This interaction disrupts lipid packing, leading to OM expansion, surface bulging, and increased permeability^5^. Colistin can then penetrate the periplasm and damage the cytoplasmic membrane, causing leakage of cellular contents and cell lysis^6^. Most identified resistance mechanisms involve modification of lipid A, reducing electrostatic interactions with colistin. In particular, plasmid-borne *mcr* genes encode phosphoethanolamine transferases that increase lipid A positive charge, thereby diminishing colistin binding affinity. These resistant genes have been reported globally in multiple Enterobacteriaceae isolates^7,8^.

In 1944, Bigger reported a subpopulation of bacteria that survived intensive antibiotic treatment, coining the term “persisters”^9^. Persistence is now defined as the ability of a small subset within a susceptible clonal population to survive bactericidal antibiotics without acquiring resistance genes or mutations^10^. These persisters are a frequent cause of treatment failure in chronic infections^11,12^. Unlike resistant cells, persisters are genetically identical to susceptible cells, making them difficult to detect using standard molecular methods. Conventional antibiotic susceptibility tests, such as the minimal inhibitory concentration (MIC) assay, cannot identify persisters, and the current standard time-kill assay only detects surviving colonies after antibiotic exposure, not persisters themselves^10,13^. A pioneering study using a microfluidic device and fluorescent-labelled *Escherichia coli* first revealed non-growing persisters under ampicillin treatment^14^. Green fluorescent protein dilution combined with flow cytometry further showed that non-growing cells survive antibiotic exposure and remain viable after antibiotic removal^15^. Unlike inheritable resistance, persisters can halt physiological activities, such as protein translation, reducing antibiotic targets while retaining the ability to resuscitate and regain susceptibility^16^. However, growth arrest alone cannot explain survival against antibiotics acting independently of growth, such as colistin targeting LPS, and little is known about morphological differences between susceptible and persistent cells.

In this study, direct stochastic optical reconstruction microscopy (dSTORM) was used to map the nanoscale distribution of LPS in Gram-negative bacteria under colistin treatment. dSTORM is a super-resolution imaging technique that achieves nanoscale resolution by precisely localising individual fluorophores, allowing computational analysis of their spatial relationship^17,18^. Here, we show that colistin-persistent *E. coli* and *Pseudomonas aeruginosa* exhibit distinct morphologies, with increased LPS clustering in persisters of both species and altered membrane lipid distribution specifically in *P. aeruginosa* persisters, compared to their susceptible counterparts. Interestingly, *mcr-1*^*+*^ resistant *E. coli* exhibited a pronounced shift in LPS distribution and a similar increase in LPS aggregation, without corresponding changes in membrane organisation. These results suggest that enhanced LPS clustering and reorganisation are associated with bacterial survival under colistin stress, indicating a shared phenotypic feature between persistence and resistance.

## Results

### Establishing an experimental framework for the isolation of persisters

**Fig. 1:**
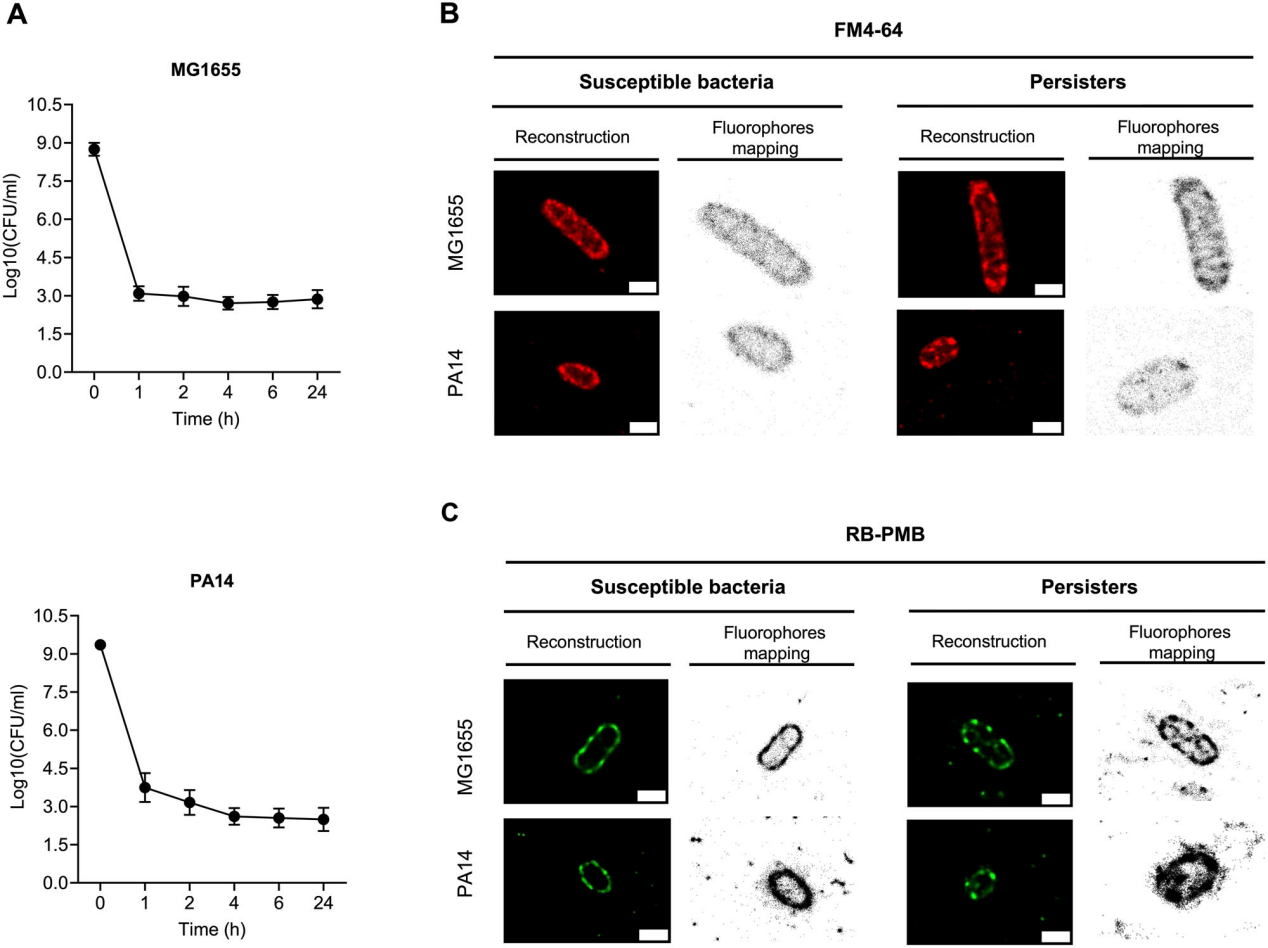
Colistin persistence and dSTORM visualisation of bacterial envelope structures. **A** Biphasic time-kill curves of Ec1655 and PA14 treated with colistin. Ec1655 was exposed to 16 μg ml^-1^ and PA14 to 64 μg ml^-1^ colistin. Bacterial survival was assessed at 0 min, 1 h, 2 h, 4 h, 6 h, and 24 h, showing a characteristic biphasic killing profile with an initial rapid decline in colony counts, followed by persistence of a small surviving population. Data represent the mean ± SD of three biological and technical replicates (*n* = 3). Reconstructed dSTORM images and computational maps of bacterial cells stained with **B** FM4-64 to visualise membrane lipids and **C** rhodamine B-labelled polymyxin B (RB-PMB) to visualise LPS distribution. Images were reconstructed from 6000 raw frames using Huygens Localizer software. Representative images of untreated susceptible cells and colistin persisters are shown for both Ec1655 and PA14. The scale bars are 1 µm.

Colistin is a potent bactericidal antibiotic used against Gram-negative bacteria. To investigate membrane architecture under colistin stress, *E. coli* MG1655 (Ec1655) and *P. aeruginosa* PA14 reference strains were selected. Susceptibility was determined by MIC assays, which measured optical density at 600 nm wavelength (OD_600nm_) after overnight growth in 96-well plates. The MIC of colistin was 1 μg ml^-1^ against Ec1655 and 4 μg ml^-1^ against PA14, confirming their colistin-susceptibility (Supplementary Fig. 1). While clinical breakpoints are commonly used for resistance classification, they are not directly comparable here; instead, the MICs provided baselines for the colistin concentrations used to assess persistence in time-kill assays. High-density stationary-phase cultures (10^9^ CFU ml^-1^) were exposed to 16 × MIC colistin (16 μg ml^-1^ for Ec1655; 64 μg ml^-1^ for PA14) (Fig. 1A). Both strains showed biphasic killing, with rapid initial declines (64.7% for Ec1655; 60% for PA14) within one hour, followed by survival of ~10^3^ CFU ml^-1^ until 24 h, indicating the presence of persisters.

Colistin compromises bacterial membrane integrity, resulting in cell lysis^6,19^. During colistin time-kill assays (16 μg ml^-1^ for Ec1655; 64 μg ml^-1^ for PA14), phase-contrast optical microscopy revealed time-dependent lysis in colistin-sensitive strains (Supplementary Fig. 2). Treatment with lytic antibiotics leads to a reduction in intact rod-shaped bacterial cells, which can be readily visualised by phase-contrast microscopy^20^. Rapid lysis at early time points highlighted colistin’s potent bactericidal activity. By 24 h, the population was dominated by cell debris, with only a small fraction of intact cells remaining, representing the persister subpopulation. Exploiting the size differences between intact and lysed bacterial cells caused by colistin, lysed debris was removed using nanopore-sized cellulose membrane filtration to enrich and collect intact persisters.

### Heterogeneous Distribution of LPS on Gram-negative Bacterial envelope

To evaluate morphological differences between colistin-susceptible and persistent cells, Ec1655 and PA14 in exponential phase were either untreated (susceptible) or exposed to their 16 × MIC of colistin to acquire persisters. Intact cells were fixed with glutaraldehyde and collected by cellulose membrane filtration. To characterise the bacterial membrane envelope and LPS, cells were stained with the lipophilic dye FM4-64, which has high affinity to the phospholipid^21^, and rhodamine B-labelled polymyxin B (RB-PMB)^22^, a fluorescent probe for LPS. Single molecule blinking events were captured by dSTORM and images were reconstructed from 6,000 frames. Each fluorophore was calculated with 20–30 nm precision (Supplementary Fig. 3) and digitally mapped for image reconstruction and spatial analysis. Reconstructed dSTORM images revealed bacterial morphology and fluorophore distribution along the cell envelope (Fig. 1B, C; Supplementary Fig. 4–8). Both Ec1655 and PA14 exhibited characteristic rod-shaped morphology, with lengths of 1–3 μm and widths of 0.25–1.0 μm. PA14 cells were generally smaller and tapered, in contrast to the more elongated, rounded shape of Ec1655. Image brightness reflected local fluorophore density, providing a visual map of spatial distribution, while sparsely labelled regions appeared dimmer due to contrast differences. While computational mapping aided visualisation, all quantitative analyses were performed using raw localisation data to ensure analytical accuracy.

In both Ec1655 and PA14, FM4-64 fluorescence predominantly localised to the cell envelope in susceptible and persistent cells, indicating membrane lipid distribution. Occasional intracellular signals suggested the presence of internalised lipids (Fig. 1B; Supplementary Fig. 5, 7). Reconstructed dSTORM images and computational maps revealed a continuous membrane signal with variable density. While susceptible cells showed uniform FM4-64 distribution, persisters displayed irregular aggregation, with distinct regions of signal enrichment and depletion. Colistin treatment also significantly reduced cell size in persisters compared to untreated cells (Supplementary Fig. 11). In both susceptible and colistin-persistent Ec1655, a spiral-like intracellular fluorescence pattern was observed (Fig. 1B; Supplementary Fig. 5). RB-PMB dSTORM imaging revealed LPS as discrete aggregates along the cell envelope, contrasting with the continuous membrane lipid distribution (Fig. 1C; Supplementary Fig. 6, 8). In susceptible Ec1655 and PA14, LPS showed an intermittent clustering pattern with alternating regions of high and low density. This distribution pattern was preserved in persister cells but with more pronounced clustering. In Ec1655 persisters, LPS aggregates appeared along the entire envelope, while in PA14, aggregation was more concentrated at the poles.

To assess the potential influence of cellulose membrane filtration on membrane and LPS distribution, untreated and unfiltered Ec1655 and PA14 cells were examined by dSTORM. The distribution patterns of membrane lipids and LPS were preserved and comparable to those observed in filtered, non-colistin-treated cells, indicating that filtration had no significant difference on persister isolation (Supplementary Fig. 4).

### Non-normal distribution of bacterial membrane lipids and LPS

**Fig. 2:**
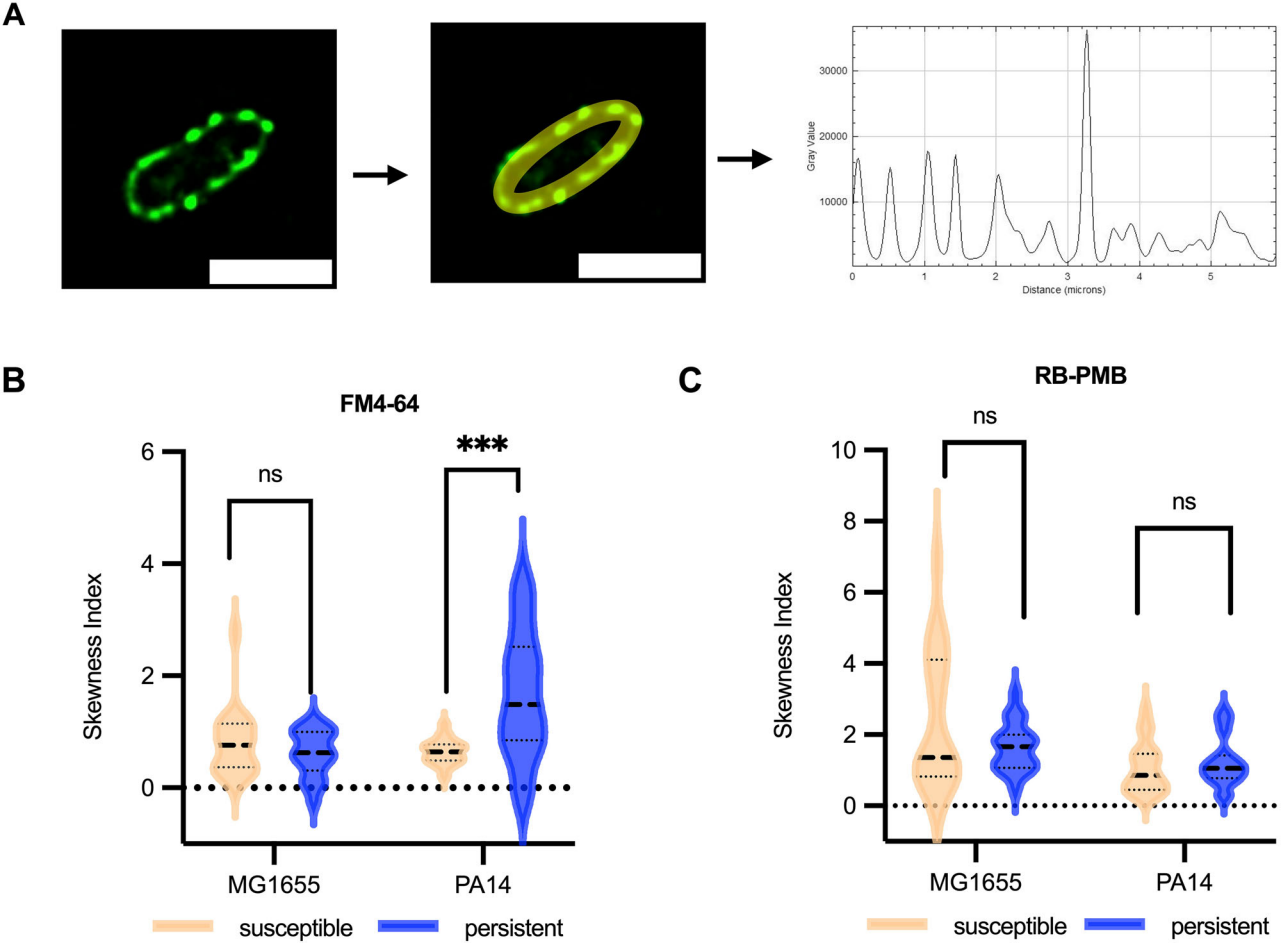
Skewed membrane distribution in susceptible and persistent *E. coli* and *P. aeruginosa.* **A** Illustration of fluorophore distribution preparation for skewness analysis. The bacterial membrane region was selected from dSTORM images. Scale bars are 2 µm. In the grey value plot, the abscissa represents the 20 × 20 nm^2^ area perimeter along the bacterial membrane, while the ordinate indicates the corresponding grey value intensity of fluorophores. Violin plots showing the skewness indices of **B** FM4-64-labelled membrane lipids and **C** RB-PMB-bound LPS along the cell envelope. Each plot includes data from 20 individual bacterial cells per condition. Plot lines indicate the first quartile, median, and third quartile. The horizontal dotted line denotes a skewness value of zero, representing a normal distribution. The significance test is an unpaired t test with Welch’s correction. NS (non-significant), *P* > 0.05; ***, *P* ≤ 0.001.

dSTORM images indicate a heterogeneous spatial distribution of LPS along the bacterial cell envelope. To evaluate distribution uniformity, skewness analysis was performed on fluorescence grey values within each 20 × 20 nm^2^ area across individual cells (Fig. 2A). Both membrane lipids and LPS in susceptible and persistent Ec1655 and PA14 displayed an overall positive skew, indicating their non-normal distribution (Fig. 2B, C), while a minority of cells showed negative skewness, reflecting intercellular variability (Supplementary Table 1). In Ec1655, membrane skewness did not differ significantly between susceptible and persistent cells. However, PA14 persisters showed a significantly increased membrane skewness and greater cell-to-cell variability compared to susceptible cells. LPS skewness remained unchanged in both species regardless of persistence status. Interestingly, the altered membrane distribution in PA14 persisters did not affect LPS distribution. This suggests that colistin persistence in different bacteria may be attributed to variations in their membrane composition leading to the difference of membrane distribution, rather than only in colistin targeted LPS.

### Colistin alters LPS and membrane lipids aggregation in colistin persistent bacteria

**Fig. 3:**
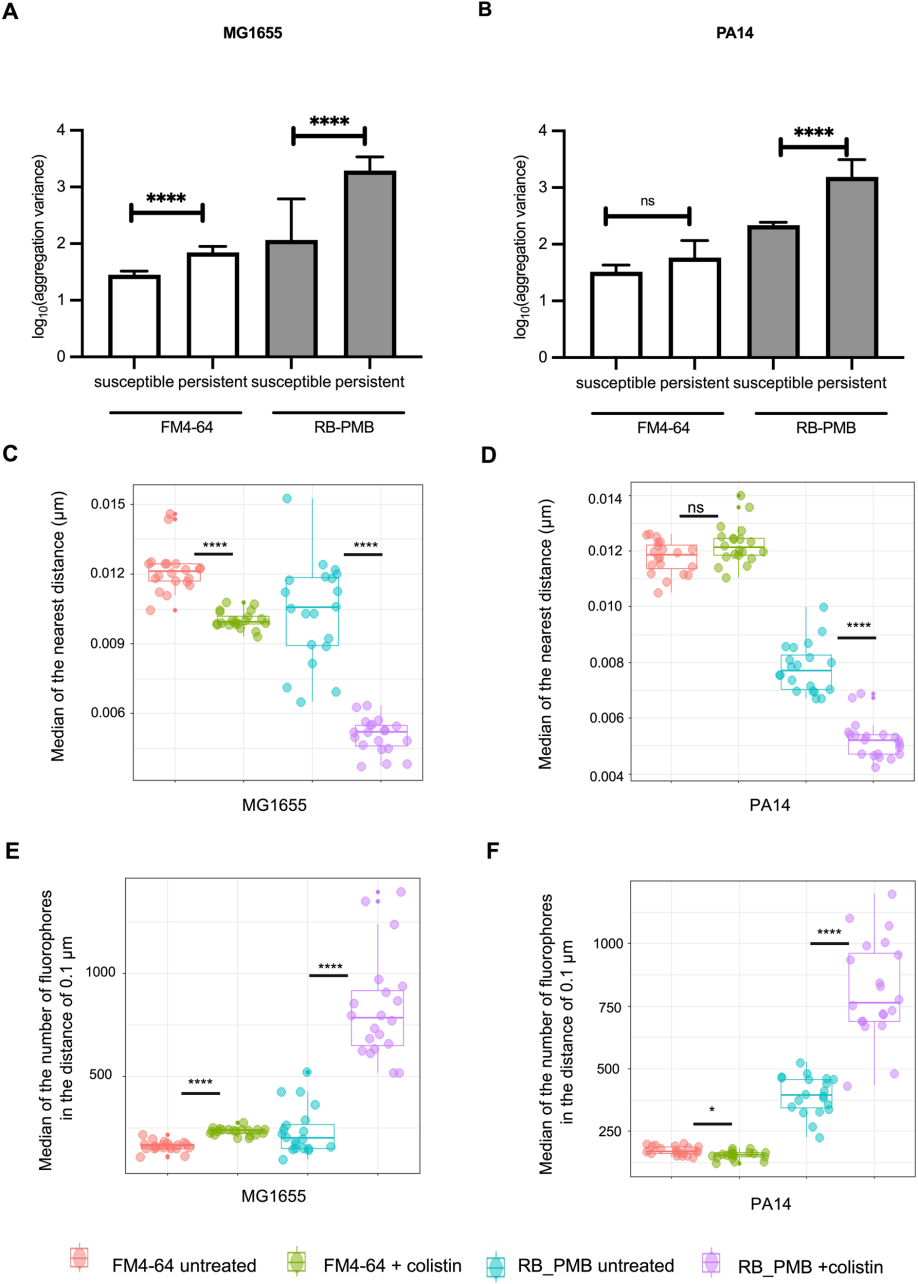
Shift of membrane molecule aggregation in response to colistin. Variance in fluorophore aggregation along the cell envelope for **A** Ec1655 and **B** PA14. The total number of fluorophores within each 20 × 20 nm^2^ pixel area along the cell perimeter was recorded. Each bar represents the median, and error bars denote 95% confidence intervals (*n* = 20 cells per condition). Statistical significance was assessed using the Mann–Whitney test. NS (non-significant) *P* > 0.05; ****, *P* ≤ 0.0001. Nearest-neighbour distance of fluorophores in untreated (susceptible) bacteria and colistin-treated (persister) bacteria of **C** Ec1655 and **D** PA14. For each fluorophore, the mean distance to its five closest neighbouring fluorophores was calculated. Fluorophore density within a 0.1 μm radius of each fluorophore in untreated (susceptible) bacteria and colistin-treated (persister) bacteria of **E** Ec1655 and **F** PA14. The number of fluorophores within a 0.1 μm radius surrounding each fluorophore centre was quantified. For **C**–**F**, each point represents a single cell (*n* = 20 cells per condition). In the box plots, the horizontal line indicates the median; box edges represent the interquartile range (IQR); whiskers extend to 1.5 × IQR, and outliers are plotted as points. Statistical significance was evaluated using the Wilcoxon test with Bonferroni correction. NS (non-significant) *P* > 0.05); *, *P* ≤ 0.05; ****, *P* ≤ 0.0001.

While reconstructed dSTORM images and computational maps revealed the heterogeneous distributions of membrane lipids and LPS in susceptible and persistent Ec1655 and PA14, a deeper understanding of their clustering behaviours and spatial organisations is essential to identify unique features associated with persistence. To quantify variability in aggregation, the variance (*σ*^*2*^) of fluorophore counts within 20 × 20 nm^2^ bins along manually annotated cell envelopes was calculated (Fig. 3A, B). This analysis was performed on twenty cells per condition. The medians of the average variance and the ratio of persistent to susceptible variance (*σ*_*p*_^*2*^*/σ*_*s*_^*2*^) were used to assess spatial heterogeneity (Supplementary Tables 2, 3). In persistent cells, membrane lipid aggregation variance exhibited strain-specific responses, while LPS variance increased significantly in both Ec1655 and PA14. In Ec1655 persisters, aggregation variance increased in both membrane lipids and LPS compared to susceptible cells (Fig. 3A), with LPS showing a larger shift (*σ*_*p*_^*2*^*/σ*_*s*_^*2*^ = 15.42) than membrane lipids (*σ*_*p*_^*2*^*/σ*_*s*_^*2*^ = 2.49). In PA14, membrane lipid variance remained unchanged, whereas LPS variance increased substantially (Fig. 3B; *σ*_*p*_^*2*^*/σ*_*s*_^*2*^ = 7.20 for LPS versus 1.93 for membrane). These results highlight distinct aggregation of membrane lipids and, in particular, LPS, underscoring topographical differences between colistin-susceptible and persistent cells.

To further characterise clustering of membrane lipids and LPS in susceptible and persistent cells, nearest-neighbour distances between fluorophores were quantified. For each fluorophore, the distances to its five closest neighbours were averaged to calculate a mean nearest-neighbour distance. The median of all mean nearest-neighbour distances was then computed per cell to summarise local spatial organisation across 20 cells per condition by fluorophore type and treatment status (Fig. 3C, D). Ec1655 persisters exhibited an average 18% reduction in membrane fluorophore spacing, indicating increased local lipid density, whereas PA14 persisters exhibited no change. Combined with variance analysis, this clustering appeared localised to specific envelope regions, enhancing aggregation variance and spatial heterogeneity. In contrast, LPS nearest-neighbour distances were significantly reduced in both Ec1655 and PA14 persisters, suggesting tight LPS clustering is a conserved feature of persistence.

To determine whether reduced nearest-neighbour distances reflected redistribution or increased local density, the number of fluorophores within defined spatial ranges (0.1–0.4 μm diameter) surrounding each fluorophore was quantified (Fig. 3E, F; Supplementary Fig. 12). Ec1655 persisters showed a significant 1.5-fold increase in lipid-tagged fluorophores across all spatial ranges compared to susceptible cells, indicating tighter membrane lipid aggregation. In contrast, PA14 persisters exhibited a slight reduction at 0.1 μm and no significant change at larger ranges, which is consistent with an unaltered membrane aggregation variance. LPS in both Ec1655 and PA14 persisters displayed a two- to threefold increase in local density across all spatial ranges, with the most pronounced enrichment within the 0.1 μm range. The numbers of fluorophores declined with increasing spatial range, indicating formation of small, tightly packed LPS clusters. These findings align with LPS variance analysis and support localised LPS enrichment as a conserved feature of persistence under colistin stress.

### Colistin alters the membrane dynamic in colistin-resistance *E. coli*

**Fig. 4:**
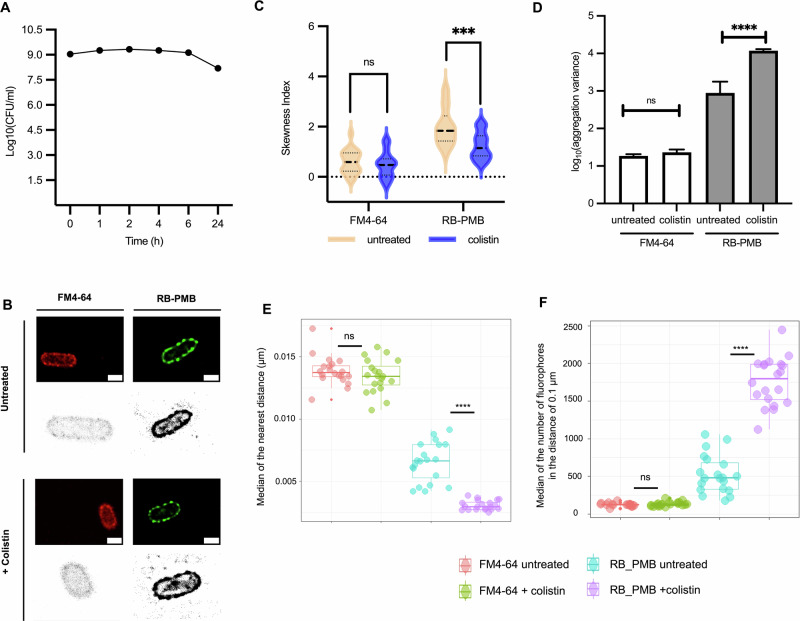
LPS redistribution and clustering in *E. coli mcr-1* resistant NCTC13846 in response to colistin. **A** Time-kill curve of Ec13846 treated with colistin at 64 μg ml^-1^. Bacterial cultures were plated at 0 min, 1 h, 2 h, 4 h, 6 h, and 24 h. Surviving colonies were counted and averaged across technical and biological replicates (mean ± SD, CFU ml^-1^, *n* = 3). **B** Reconstructed dSTORM images and computational maps of untreated and colistin-treated Ec13846 stained with FM4-64 (membrane lipids) and RB-PMB (LPS). Images were reconstructed from 6000 raw frames using Huygens Localizer software. The scale bars are 1 µm. **C** Violin plots showing skewness indices of membrane lipids and LPS distributions under untreated and colistin-treated conditions. Each plot represents data from 20 single cells. Lines denote the first quartile, median, and third quartile. The horizontal dotted line indicates a skewness of zero. Statistical analysis was performed using an unpaired t-test with Welch’s correction. NS (non-significant), *P* > 0.05; ***, *P* ≤ 0.001. **D** Increased variance in LPS aggregation following colistin treatment. Bar heights indicate medians, with error bars showing the 95% confidence intervals. Statistical analysis was performed using the Mann-Whitney test. NS (non-significant), *P* > 0.05; ****, *P* ≤ 0.0001. **E** Decreased nearest-neighbour distances of LPS after colistin exposure. For each fluorophore, the mean distance to its five closest neighbouring fluorophores was calculated. **F** Increased LPS density within a 0.1 μm radius around each fluorophore. The number of fluorophores within a 0.1 μm radius surrounding each fluorophore centre was quantified. In **E**, **F**, each point represents a single cell (*n* = 20). In the box plots, the horizontal line indicates the median; box edges represent the interquartile range (IQR); whiskers extend to 1.5 × IQR, and outliers are plotted as points. Statistical significance was evaluated using the Wilcoxon test with Bonferroni correction. NS (non-significant) *P* > 0.05); ****, *P* ≤ 0.0001.

Given the alterations in membrane lipids and LPS distribution observed in colistin persisters, we next investigated whether similar dynamics occur in resistant bacteria. *E. coli* NCTC 13846 (Ec13846), carrying the *mcr-1* gene^23,24^, exhibited high-level resistance with a colistin MIC of 16 μg ml⁻¹ (Supplementary Fig. 1). Following exposure to 64 μg ml⁻¹ colistin, Ec13846 maintained colony counts with only an 11% reduction after 24 h (Fig. 4A) and preserved cellular integrity throughout treatment with 64 μg ml^-1^ colistin (Supplementary Fig. 2). dSTORM imaging revealed no major differences in membrane or LPS patterns between treated and untreated cells (Fig. 4B; Supplementary Figs. 9, 10). As expected, both membrane lipids and LPS showed overall positively skewed, non-normal distribution along the envelope (Fig. 4C). Notably, while membrane skewness remained unchanged following treatment, LPS skewness decreased substantially (mean skewness index from 1.99 to 1.23), indicating a shift toward a more uniform distribution. This LPS reorganisation suggests that resistance may involve not only chemical modification reducing colistin affinity, but also structural rearrangement of LPS around the cell envelope.

In colistin-treated Ec13846, LPS aggregation variance increased significantly compared to untreated cells (*σ*_*c*_^2^*/σ*_*u*_^*2*^ = 13.24), while membrane lipid variance remained relatively unchanged (*σ*_*c*_^*2*^*/σ*_*u*_^*2*^ = 1.23), highlighting distinct organisational responses of LPS and membrane lipids to colistin pressure (Fig. 4D; Supplementary Table 4). Consistent lipid spacing and stable FM4-64-labelled lipid counts within defined areas further confirmed that membrane lipid organisation was maintained following treatment (Fig. 4E, F; Supplementary Fig. 12; Table 4). Clustered LPS in colistin-treated Ec13846, characterised by a 53% reduction in nearest-neighbour distances and a two- to threefold increase in LPS counts within local areas, supports the notion that tight LPS aggregation is a common bacterial response to colistin stress. Unlike the membrane reorganisation observed in Ec1655 persisters, the changes in LPS clustering and distribution in Ec13846 occurred without accompanying membrane restructuring, suggesting mechanistic differences between resistance in Ec13846 and persistence in Ec1655.

## Discussion

LPS is a major structural component of the outer leaflet of the Gram-negative bacterial OM, forming a permeable barrier that protects cells from external stresses. The structure and composition of LPS vary between bacterial species, contributing to differences in OM permeability^25^. In *P. aeruginosa*, the addition of positively charged aminoarabinose to lipid A phosphate groups reduces OM permeability by 12–100 fold compared to *E. coli*, enhancing *P. aeruginosa* intrinsic resistance to a wide range of antibiotics^26,27^. Consistent with this, *P. aeruginosa* PA14 exhibited a fourfold higher colistin MIC than *E. coli* MG1655 in our study, confirming greater susceptibility of *E. coli* to colistin. However, MIC values did not correlate with persistence; both strains displayed persistence, with a surviving subpopulation after high-dose colistin (16 × MIC) exposure in time-kill assays.

The low abundance of persister cells within a predominantly killed population presents a significant technical challenge for their isolation and analysis. Techniques such as microfluidic devices and flow cytometry have been used to study persisters at the single-cell level; however, the fluorescent pre-labelling required for these methods is incompatible with dSTORM imaging. To overcome this, we leveraged colistin-induced lysis of susceptible cells and isolated persisters based on size and cell integrity. Preliminary attempts to isolate intact cells via flow cytometry were hindered by dilution effects from the sheath fluid, complicating downstream microscopy. We therefore adopted a conventional filtration and centrifugation method, previously used to enrich *E. coli* persisters after β-lactam antibiotics exposure by removing filamented and lysed cells for microscopic or proteomic analysis^28–30^. This approach effectively removed lysed debris while preserving cell morphology, with no significant difference compared to unfiltered samples in dSTORM imaging (Supplementary Fig. 4).

Super-resolution dSTORM enables nanoscale localisation and visualisation of molecular organisation at the single-cell level, providing insights into bacterial structure and dynamics^17,18^. Fluorescent antibiotic probes have likewise proven valuable for probing antibiotic effects on both bacteria and host cells^31^. In both *E. coli* and *P. aeruginosa*, FM4-64 predominantly localised to the cell envelope, marking membrane lipid distribution regardless of susceptibility or persistence. Intracellular FM4-64 signals were also detected, despite generally being considered impermeable in Gram-negative bacteria, reflecting the high sensitivity of dSTORM rather than compromised membranes. Interestingly, susceptible and colistin-persistent Ec1655 cells exhibited a spiral-like intracellular fluorescence pattern, reminiscent of division-associated membrane structures observed in *Bacillus subtilis*^32^. The poles of rod-shaped bacteria display unique curvature-driven properties that promote lipid enrichment. Lipids such as cardiolipin preferentially localise to these high-curvature regions, forming functional clusters through curvature-mediated mechanisms^33,34^. Consistently, our dSTORM imaging revealed polar enrichment of membrane lipids in both species.

Compared to membrane lipids, LPS exhibited greater difference in aggregation and intermittent distribution between susceptibility and persistence in both *E. coli* and *P. aeruginosa*. Studies have shown that incorporating LPS into phospholipid liposomes leads to the formation of large domains^35^, and atomic force microscopy (AFM) imaging reveals that LPS clusters on the *E. coli* surface can dynamically merge, grow, and split apart^36^. A recent sub-diffraction microscopy study further highlighted the clustered nature of LPS in *E. coli*^37^. Aligned with these findings, our dSTORM images indicate that LPS in both *E. coli* and *P. aeruginosa* is not evenly distributed but organised into distinct clusters.

To assess the uniformity of the LPS and membrane lipid distributions along the bacterial cell envelope, we calculated the skewness index from the grey values of fluorophore signals. No zero values were detected for either membrane lipids or LPS, indicating their continuous presence across the envelope. A positive skewness reflected clustered signals within 20 × 20 nm^2^ regions, where the mean grey value exceeded the median; negative skewness indicated dispersed distributions, and a skewness of zero corresponded to a normal distribution. Skewness analysis revealed non-normal distributions of both membrane lipids and LPS in *E. coli* and *P. aeruginosa*. In *E. coli* persisters, skewness of membrane lipids and LPS was comparable to those in susceptible cells. In contrast, *P. aeruginosa* persisters showed significantly increased membrane lipid skewness and higher cell-to-cell variability, while LPS skewness remained unchanged. These differences may reflect species-specific variations in membrane lipid composition and biosynthetic pathways^38^, influencing envelope organisation in response to colistin stress.

Skewness of the overall distributions along the cell envelope may not fully capture localised organisations of membrane lipid and LPS. Indeed, local clustering analysis revealed that molecular aggregation patterns did not always correspond to the global distribution observed through skewness. In *E. coli* persisters, the local membrane lipid density increased compared to susceptible cells, but without significantly altering skewness, whereas in PA14 persisters, membrane density remained stable, although overall skewness increased. These findings highlight distinct topographical effects of colistin on bacterial membranes, suggesting that the biophysical mechanisms of persistence differ between strains. Further analysis showed significant reductions in LPS nearest-neighbour distances and increased local LPS density in both *E. coli* and *P. aeruginosa* persisters, indicating that the formation of tightly clustered LPS is a conserved feature of persistence, potentially enhancing survival against colistin-mediated disruption.

Our findings on LPS and membrane rearrangement in colistin-treated cells echo previous reports suggesting that colistin modulates membrane fluidity^39,40^. However, prior studies were conducted at MIC or sublethal concentrations, which may not reflect the cellular responses of persisters under high-dose exposure. These studies, however, reported conflicting effects: Ginez et al. observed increased membrane fluidity following polymyxin treatment^39^, while Manioglu et al. proposed that colistin–LPS interactions induce rigid crystalline structures, leading to membrane solidification^40^. Our data demonstrate that membrane lipids and LPS undergo spatial reorganisation under colistin stress, raising the question of whether such rearrangement is compatible with membrane solidification. Supporting this, Benn et al. proposed that the crystalline structures detected by AFM represent outer membrane protein lattices, with colistin-bound LPS enhancing their visualisation^41^. Whereas AFM captures highly localised nanoscale features, our dSTORM imaging captures a broader view of membrane architecture across whole cells. We propose that persister cells dynamically remodel membrane components to maintain envelope integrity, supporting survival under antibiotic stress. These findings reinforce the concept that membrane plasticity is a key determinant of bacterial persistence.

Phosphoethanolamine transferase, encoded by *mcr* genes, modifies LPS to reduce colistin binding affinity, thereby conferring resistance^7^. Based on this mechanism, membrane or LPS rearrangement was not anticipated in colistin-resistant *E. coli* (*mcr-1*^*+*^). As expected, membrane distribution, lipid aggregation variance, spacing, and overall lipid counts remained unchanged, indicating that colistin did not perturb membrane structure, unlike the disruptions observed in *E. coli* persisters. Unexpectedly, the LPS distribution in colistin-treated *mcr-1*^*+*^ *E. coli* shifted toward a more normal pattern (Fig. 4C), accompanied by shortened nearest-neighbour distances and increased local LPS density, consistent with LPS clustering. Despite reduced binding affinity, this reorganisation suggests that *mcr-1*^*+*^ cells may either actively respond to colistin stress or passively adapt through LPS-membrane dynamics.

Membrane stability is maintained through complex networks of sensing and response pathways that regulate molecular transport and remove mislocalised components^42^. Our study demonstrates that, compared to susceptible cells, colistin persisters in Gram-negative bacteria exhibit distinct morphological changes in LPS and membrane lipid distribution across the cell envelope. LPS clustering appears to act as a stress-mitigation strategy, supporting survival under colistin exposure in both persistence and resistance. Given this complexity, targeting membrane stability offers a promising avenue for enhancing antibiotic efficacy. One compelling example is the antimalarial compound mefloquine, which reduces bacterial membrane fluidity and synergistically enhances the activity of polymyxin antibiotics^43^. Our findings advance understanding of bacterial persistence and resistance by revealing membrane organisation under colistin stress. This study provides a first characterisation of phenotypic responses, establishing a foundation for exploring how membrane reorganisation contributes to persistence and resistance. By highlighting these shared features, our work opens new avenues for investigating underlying mechanisms and guiding future studies on the molecular basis of bacterial survival under antibiotic stress.

## Methods

### Bacterial strains and culture conditions

*E. coli* K-12 MG1655, *E. coli* NCTC 13846, and *P. aeruginosa* PA14 were routinely cultured in Luria Broth (LB, Difco 244620) overnight in a shaker incubator at 37 °C and 200 rpm or on 1.5% (w/v) agar plate at 37 °C incubation.

### Antibiotic susceptibility test

The minimum inhibitory concentration (MIC) was determined by using microdilution in a 96-well microtiter plate format^44^. Briefly, the colistin concentrations with two times dilution are prepared from 0.5 to 64 μg ml^−1^. The bacteria overnight cultures were diluted to OD_600nm_ = 0.01 as the initial inoculum. Three controls were included in each plate including LB only, LB with 64 μg ml^−1^ colistin, and LB with bacterial inoculum. Plates were incubated at 37 °C for 24 h, and the optical density at 600 nm was measured in technical triplicates for each condition using spectrophotometry. The MIC was determined based on the turbidity with increasing OD, suggesting the growth of bacteria. All experiments were performed in three independent biological replicates.

### Time killing assay

The overnight culture was diluted in fresh LB medium (100 μl in 50 ml) in flask and incubated at 37 °C and with shaking at 200 rpm for an additional 7 h, allowing bacteria to achieve the early stationary phase. After 7 h incubation, 100 μl bacterial culture was harvested for determining the colony forming unit (CFU) as the reference of the initial bacterial numbers of the killing assay, and the relevant concentrations of colistin was added into the bacteria culture. The concentration of colistin for *E. coli* K-12 MG1655 was 16 μg ml^−1^, and for both *E. coli* NCTC 13846 and *P. aeruginosa* PA14 it was 64 μg ml^−1^. The colistin killing was then assessed at defined time points (0.5, 1, 1.5, 2, 4, 6, 24 h) by taking 100 μl culture from the killing incubation for series dilutions and spread onto LB agars for the CFU count. All experiments were performed in three independent biological replicates.

### Preparation of bacteria samples for microscopy

Bacteria in the early stationary phase were prepared as previously described, and three groups of bacterial samples were processed for dSTORM microscopy. For the first filtration control group, 1 ml of bacterial culture, without colistin treatment and without filtration, was aliquoted for glutaraldehyde fixing (see below). For the second group of bacteria, which were not treated with colistin but underwent filtration, the bacterial culture was diluted 10 × with Hanks’ Balanced Salt Solution (HBSS, pH 7.0–7.4, osmolality 260–290 mOSm/kg, without calcium and magnesium divalent; Thermo Fisher Scientific REF88284), fixed, and then filtered through a 0.2 μm polyethersulfone membrane (Thermo Fisher Scientific 564-0020). After filtration, sterile loops were used to extensively scrape the membrane to collect the filtered bacteria in 10 ml of HBSS. The scraped bacteria were then subjected to an additional filtration step using Amicon cellulose filters (100 kDa MWCO, Sigma UFC910024) and centrifuged at 3,800 × *g*_n_ for one hour. The remaining bacteria were then resuspended in 1 ml HBSS, collected from the Amicon filters, and further washed with fresh HBSS by centrifuging at 14,000 × *g*_n_ for 2 min. For the third group, bacteria were treated with lethal concentrations of colistin for 24 h at 37 °C with shaking at 200 rpm, fixed, and collected by filtration, as previously described. The fixation of bacterial samples was performed using 2.5% (v/v) glutaraldehyde (Bangslabs AA012) for 1 h at room temperature, followed by two washing steps with HBSS (14,000 × *g*_n_ for 2 min). The fixed cells were 10 × diluted in HBSS for further fluorescent staining and stored at 4 °C.

The fixed bacteria in HBSS were centrifuged, pelleted, and resuspended in dSTORM buffer (100 mM mercaptoethylamine (MEA), 20 mM sodium sulfite, 2 mM cyclooctatetraene (COT) in HBSS). The fixed bacteria were then stained with 0.55 μg ml^−1^ rhodamine B labelled polymyxin B (excitation/emission 550–560/590–610 nm, Sigma SBR00036) for 8 min at room temperature. This concentration was chosen to optimise fluorophore blinking for dSTORM, ensuring sufficient single-molecule events for reliable reconstruction and precise localisation. For membrane staining, the fixed bacteria were incubated with 5 μg ml^−1^ FM4-64 dye (excitation/emission 515/640 nm, Thermo Fisher Scientific, T13320) for 4 min at room temperature. This concentration was chosen based on previous super-resolution microscopy work in *P. fluorescens*^45^. The stained bacterial samples were then washed with dSTORM buffer and mounted on microscopic glass slides with 1.5% (w/v) agarose. The coverslips (VWR, No. 1.5, 631-0138) were plasma-cleaned at Hi mode (Harrick Plasma) prior to use.

### dSTORM image acquisition and processing

Super resolution images were recorded on a custom build dSTORM using a Nikon Ti microscope base equipped with the appropriated fluorescence filter cubes, 488 nm laser, 561 nm laser, oil-immersion 100 × NA 1.4 objective, Kinesis KDC101 motor controller, and a high speed Photometrics Evolve Delta EMCCD camera^46^. Imaging was performed under near-Total Internal Reflectance Fluorescence Highly Inclined Laminated Optical sheet (TIRF/HILO) conditions^47^, using a translatable lens in the excitation path. Samples were placed using a manual translation stage. Fluorescent molecule blinking series were acquired for 6,000 frames under a continuous 20 kW/cm^2^ laser. The acquired data (ND2 file) were reconstructed and rendered using Huygens Localizer software (Scientific Volume Imaging, Netherlands). The corresponding localisation csv files were extracted from the Localizer software, and the data in Cartesian coordinates were analysed using RStudio^48^.

Spatial resolution of the dSTORM images was measured similarly to previously described^49^. In brief, image data were tracked using bespoke tracking code which linked localisations into trajectories over time^50^. As cells are fixed, localisations should be stationary over time so any variance in their position represents error and can be used to measure the spatial precision of the dSTORM reconstructions. Thousands of localisations were tracked over time for each dye used (Supplementary Fig. 3). To obtain sufficient data for quantitative analysis, more than 20 individual cells per condition were examined across biological replicates in dSTORM, in line with previous super-resolution microscopy studies (e.g., ~10 cells per strain^51^ or 35 cells in total^52^).

### Analysis of bacterial membrane lipids and LPS distribution

Bacteria membrane regions were manually delineated in FIJI^53^ using the segmented line tool. A 20 × 20 nm^2^ area along the segmented line was determined. The grey values within this region were extracted to represent the density of the fluorescence at each pixel along the membrane regions. The skewness of the grey value distribution for each single cell was calculated based on the following formula:

1 $${Skewness}=\frac{n}{\left(n-1\right)\left(n-2\right)}\mathop{\sum }\limits_{i=1}^{n}{\left(\frac{{x}_{i}-\bar{x}}{s}\right)}^{3}$$

where, $n$ is the sample size, ${x}_{i}$ is the i-th value in the dataset, $\bar{x}$ is the mean, and $s$ is the standard deviation.

Skewness indices were visualised using violin plots and statistical significance was determined using an unpaired t-test with Welch’s correction.

### Analysis of clustering behaviour and spatial organisation

Quantitative analysis was performed in FIJI and R to assess membrane lipid and LPS clustering behaviours and spatial organisations, including aggregation variance, nearest-neighbour distances, and fluorophore densities.

To define membrane regions, polygon ROIs were manually drawn around each cell’s membrane in FIJI using the polygon ROI drawing tool, ensuring consistent segmentation across all images. These membrane ROIs were saved as zip files and subsequently processed by a custom ImageJ macro (3_Extract_Membrane_Points.ijm) to extract coordinate points of pixels within the defined membrane regions. The resulting coordinates were then imported in RStudio using the script (4_Membrane_Analysis.R). The number of fluorophores in each 20 × 20 nm^2^ bin was counted. The variance (*σ*^*2*^) of these counted fluorophores in each cell were calculated, providing a measure of spatial heterogeneity in local molecule aggregation. The calculation was based on:

2 $${\sigma }^{2}=\frac{1}{N}\mathop{\sum }\limits_{i=1}^{N}{\left({x}_{i}-\bar{x}\right)}^{2}$$

where, $N$ is the total number of spatial bins, ${x}_{i}$ is fluorophore count in the i-th bin, $\bar{x}$ is the mean.

The median of the variances across a total of twenty bacterial cells was calculated for each experimental condition of each strain. Statistical significance was determined using Mann-Whitney test. Detailed methodological steps, including the full code used for image processing are available at Github repository (see *Data Availability*).

To analyse nearest-neighbour distances and densities of fluorophores, dSTORM images were opened in FIJI, and coordinate systems aligned between the image and corresponding dSTORM csv files containing signal coordinates. Individual cells within each image were segmented manually using the rectangular ROI drawing tool, and their coordinates extracted using a custom ImageJ macro (1_Extract_Coordinates_from_Image_ROIs.ijm). The macro was designed to extract the XY coordinates that define a small region sufficient to enclose an entire single bacterial cell. These coordinates from each dSTORM reconstructed image were then imported into RStudio for further analysis via the script (2_Multi_Cell_Analysis.R). These coordinates were used to identify which fluorophores, extracted from dSTORM data, belonged to the same individual cell, as a single image often contained multiple bacteria.

Within R, XY coordinates of fluorophores were organised by cell, and spatial relationships between them were quantified. Specifically, we measured the number of spots within concentric circles with radii of 0.1 μm, 0.2 μm, 0.3 μm, and 0.4 μm around each spot, as well as the distance to its five closest neighbours (in μm). These metrics were used to assess membrane lipid and LPS clustering behaviours and spatial organisations. Individual cell data were exported as csv files. Finally, all individual cell datasets were combined into a single dataframe for visualisation and statistical analysis. Membrane density maps were generated using ggplot2. Statistical significance was determined using Wilcoxon tests with Bonferroni correction for multiple comparisons. Detailed methodological steps, including the full code used for image processing and data analysis, are available in the supplementary information and at Github repository (see *Data Availability*).

## Supplementary information

LPSRemodelling_Wang_Suppl_v2

## Data Availability

Statistical data acquired by microbiological and dSTORM experiments for preparing figures and supplementary information were deposited at the Figshare open access repository (https://figshare.com/s/aefe77e1a8212e81bac5). Other data supporting the findings of this study, such as raw dSTORM recording data, are available from the corresponding author upon reasonable request. ImageJ Macro code and R Code for image processing/analysis is available on GitHub (https://github.com/NCL-ImageAnalysis/LPS-Distribution-in-Colistin-Persisters-Differs-from-Susceptible-Bacteria/tree/main).
