# Supplementary material for: Lipopolysaccharide clustering in colistin persistent and resistant bacteria
Source: NPJ Antimicrob Resist. 2025 Oct 27;3:88. doi: 10.1038/s44259-025-00158-4 (PMC12559395; doi:10.1038/s44259-025-00158-4)
Supplement: Supplementary file 1 — LPSRemodelling_Wang_Suppl_v2 [file 44259_2025_158_MOESM1_ESM.pdf]

# Supplementary information

## Lipopolysaccharide clustering in colistin persistent and resistant bacteria

Fengyi Wang[1,2], George Merces[2,3], Dominic Alderson[2], Adam J. M. Wollman[2], Mark Geoghegan[4], Chien-Yi Chang[1,2]*

[1]School of Dental Sciences, Faculty of Medical Sciences, Newcastle University, Newcastle Upon Tyne, NE2 4BW, UK

[2]Biosciences Institute, Faculty of Medical Sciences, Newcastle University, Newcastle Upon Tyne, NE2 4HH, UK

[3]Image Analysis Unit, Faculty of Medical Sciences, Newcastle University, Newcastle Upon Tyne, NE2 4HH, UK

[4]School of Engineering, Newcastle University, Newcastle Upon Tyne, NE1 7RU, UK

*Corresponding author: Chien-Yi Chang

**Email:** chienyi.chang@newcastle.ac.uk

**Keywords:** Persistence, lipopolysaccharide, bacterial envelope, mcr-1, dSTORM

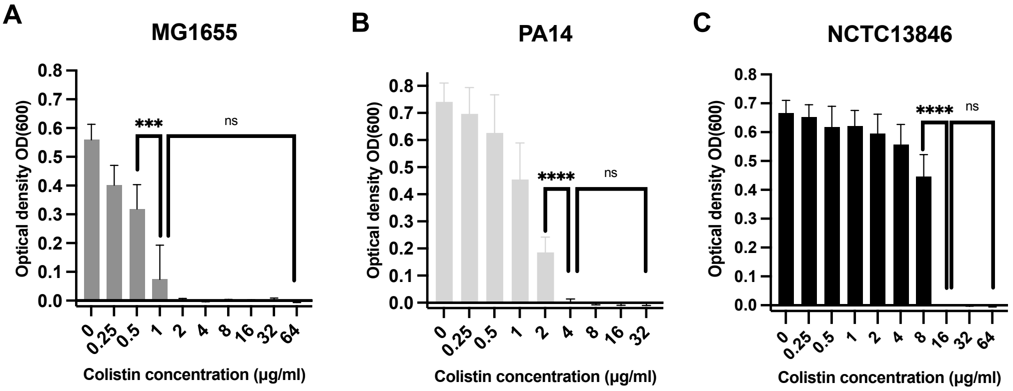


**Supplementary Figure 1. Determination of minimum inhibitory concentration (MIC) of colistin for (A) *E. coli* colistin sensitive MG1655, (B) *P. aeruginosa* PA14, and (C) *E. coli* colistin resistant NCTC13846.** Bacteria growth with colistin were assessed as measured by $OD_{600nm}$ readings of overnight bacterial growth in the 96-well microtiter plate. Data were blanked by LB medium only. The MIC was defined as the lowest colistin concentration at which bacteria growth exhibited no statistically significant difference compared to higher concentration but showed a significant difference when compared to the next lower colistin concentration. Three biological repeats were performed (n = 3). Error bar indicates standard deviation of the mean. Statistical significance calculated by unpaired t test. NS (non-significant), $P > 0.05$; ***, $P \leq 0.001$; ****, $P \leq 0.0001$.

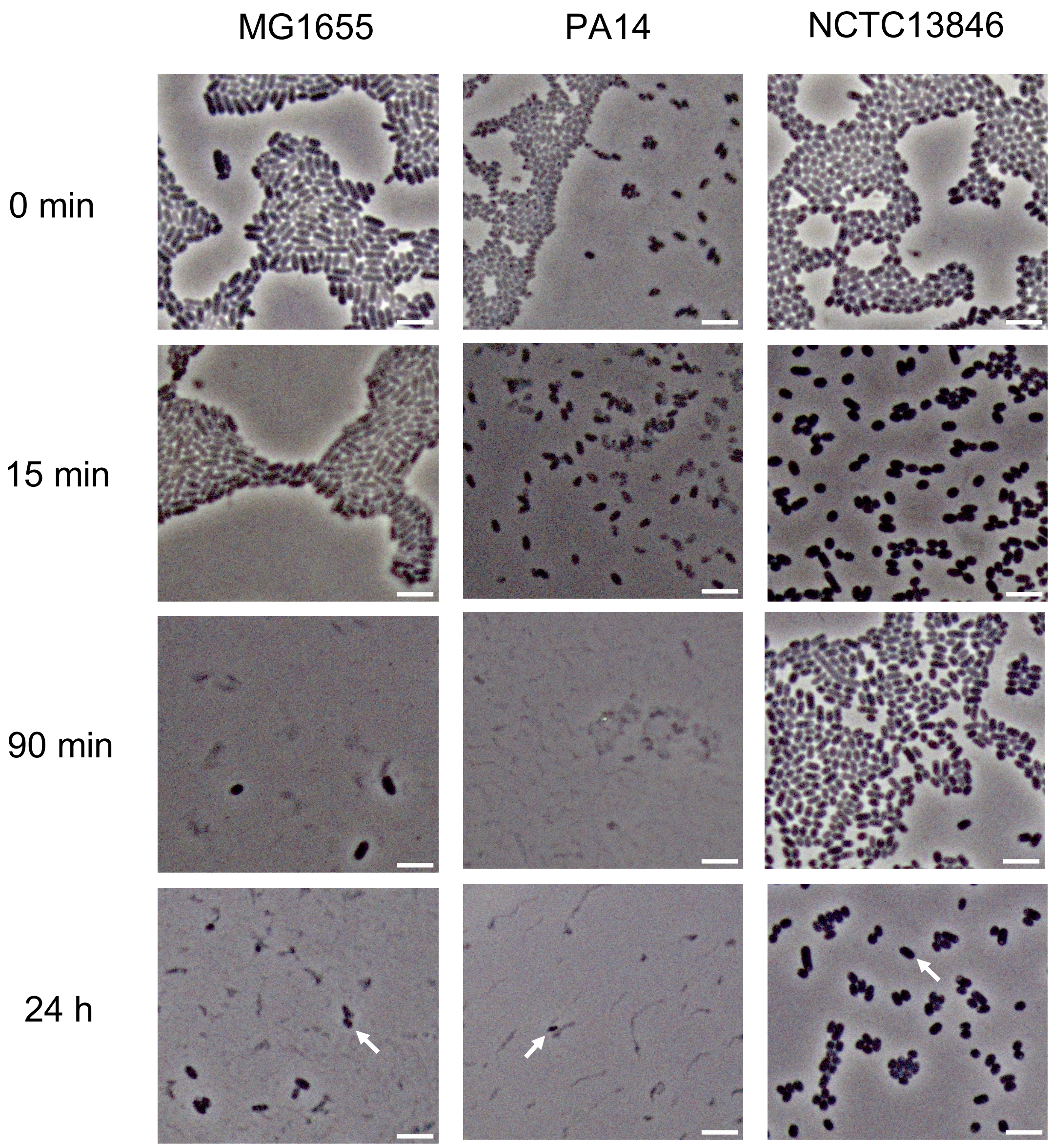


**Supplementary Figure 2. Bacteria killed by colistin observed over time with phase contrast optical microscopy.** Following the time killing assay of bacteria (16 μg $ml^{-1}$ for *E. coli* MG1655; 64 μg $ml^{-1}$ for *P. aeruginosa* PA14, 64 μg $ml^{-1}$ for *E. coli* NCTC13846), bacteria were observed at 0, 15 min, 90 min, and 24 h time points. White arrows indicate examples of intact bacterial cells maintaining cellular integrity in colistin treatments. The scale bars are 4 μm.

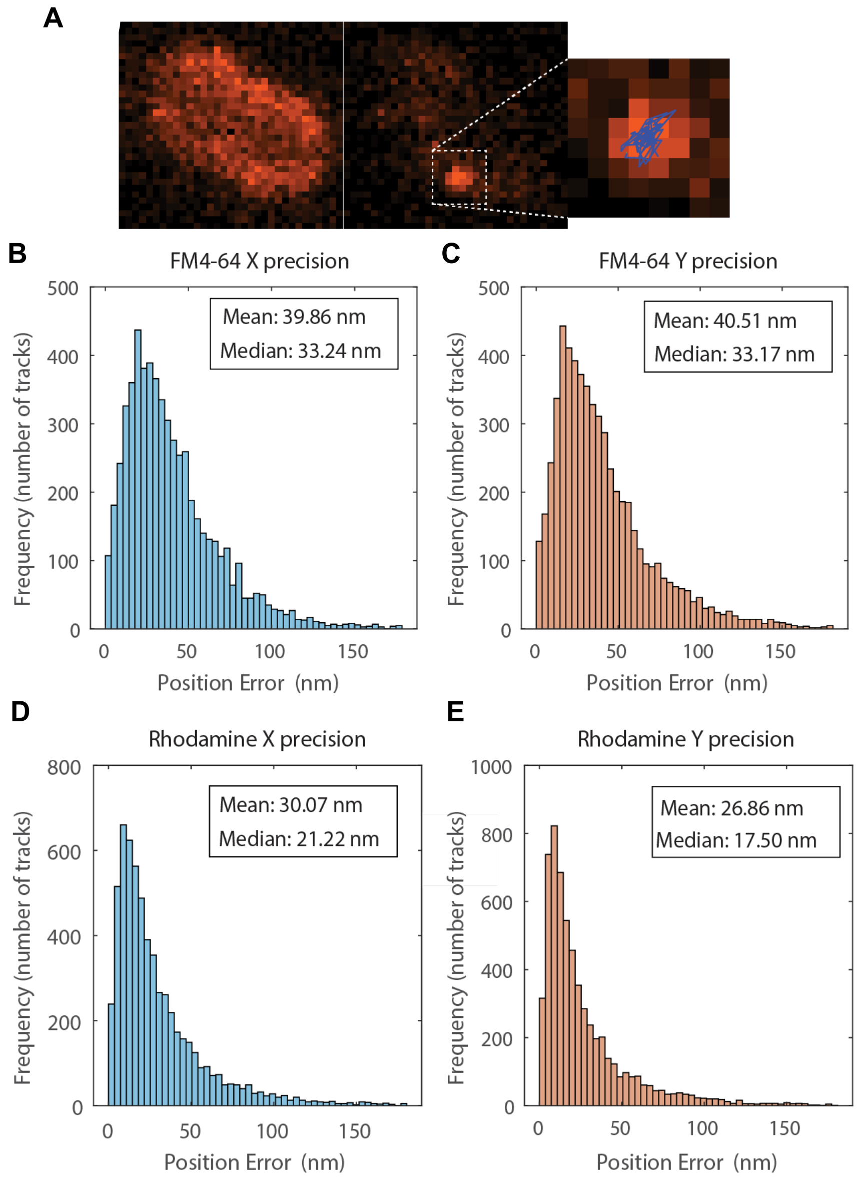


**Supplementary Figure 3. Localisation precision of dSTORM imaging.** (**A**) Illustration of tracking fluorophore foci over time to calculate the localisation precision. As cells are fixed, foci should be stationary between frames so the standard error in their position is a measure of dSTORM spatial resolution. **(B)** to **(E)** Histograms of foci position standard error of the mean for FM4-64 and RB-PMB labelled bacterial cells.

**Supplementary Figure 4 to 10** display the reconstructed dSTORM images of bacteria (*Escherichia coli* MG1655, *Pseudomonas aeruginosa* PA14, and *Escherichia coli* NCTC13846) stained with FM4-64 (membrane lipids) or RB-PMB (LPS) under respective experimental conditions. Each condition was imaged independently. Bacteria in Supplementary Fig. 4 were not treated with colistin, nor centrifuged through nanopore-sized cellulose membrane filtration. Three reconstructed dSTROM images were obtained for each condition. Bacteria samples in Supplementary Fig 5–10 has been centrifuged through filtration membrane. Each condition has been displayed nine reconstructed dSTORM images. The scale bars are 2 μm.

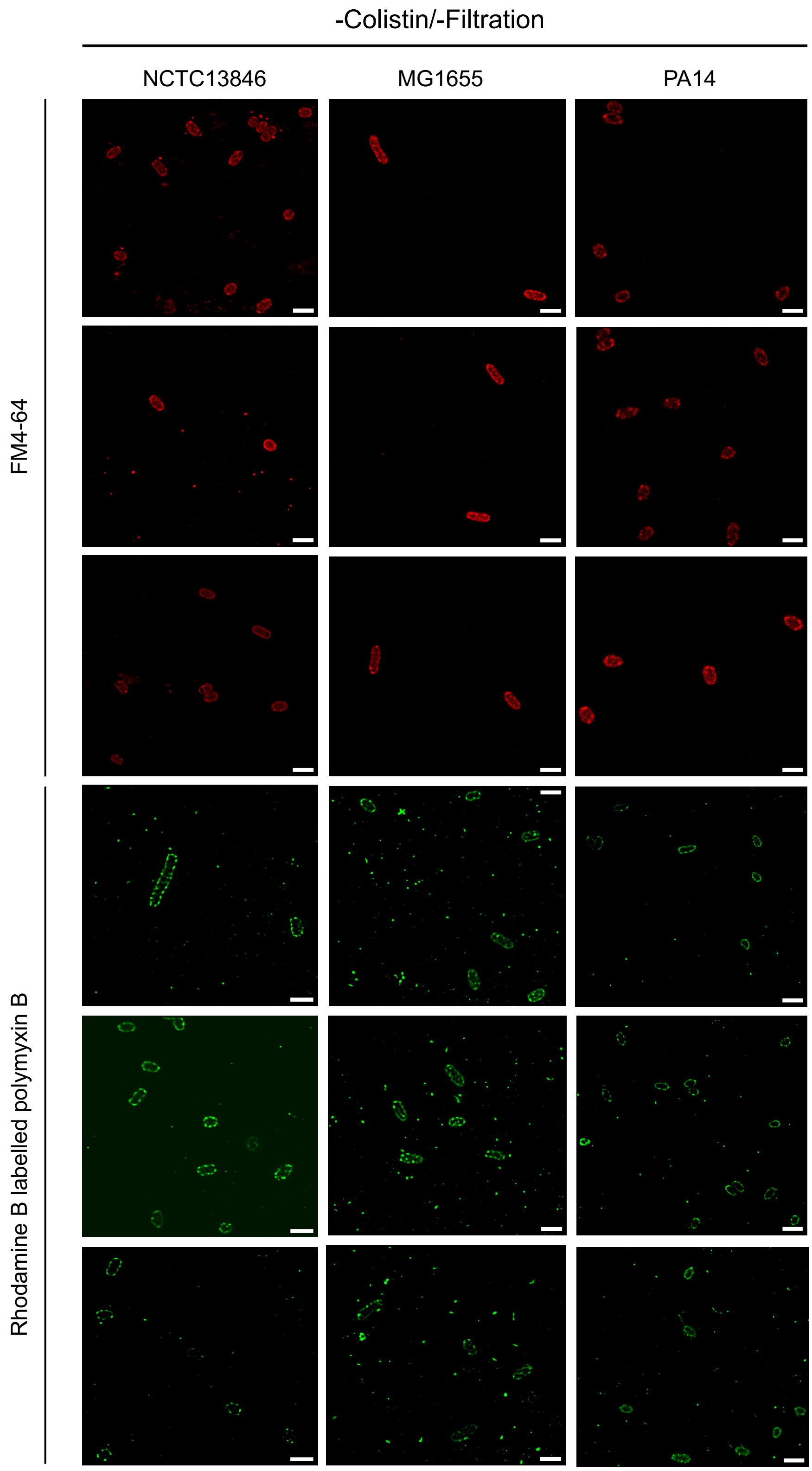


**Supplementary Figure 4. Visualisation of FM4-64-labelled membrane lipids and RB-PMB-bound LPS in three bacterial strains without colistin, centrifugation and filtration.** The scale bars are 2 µm.

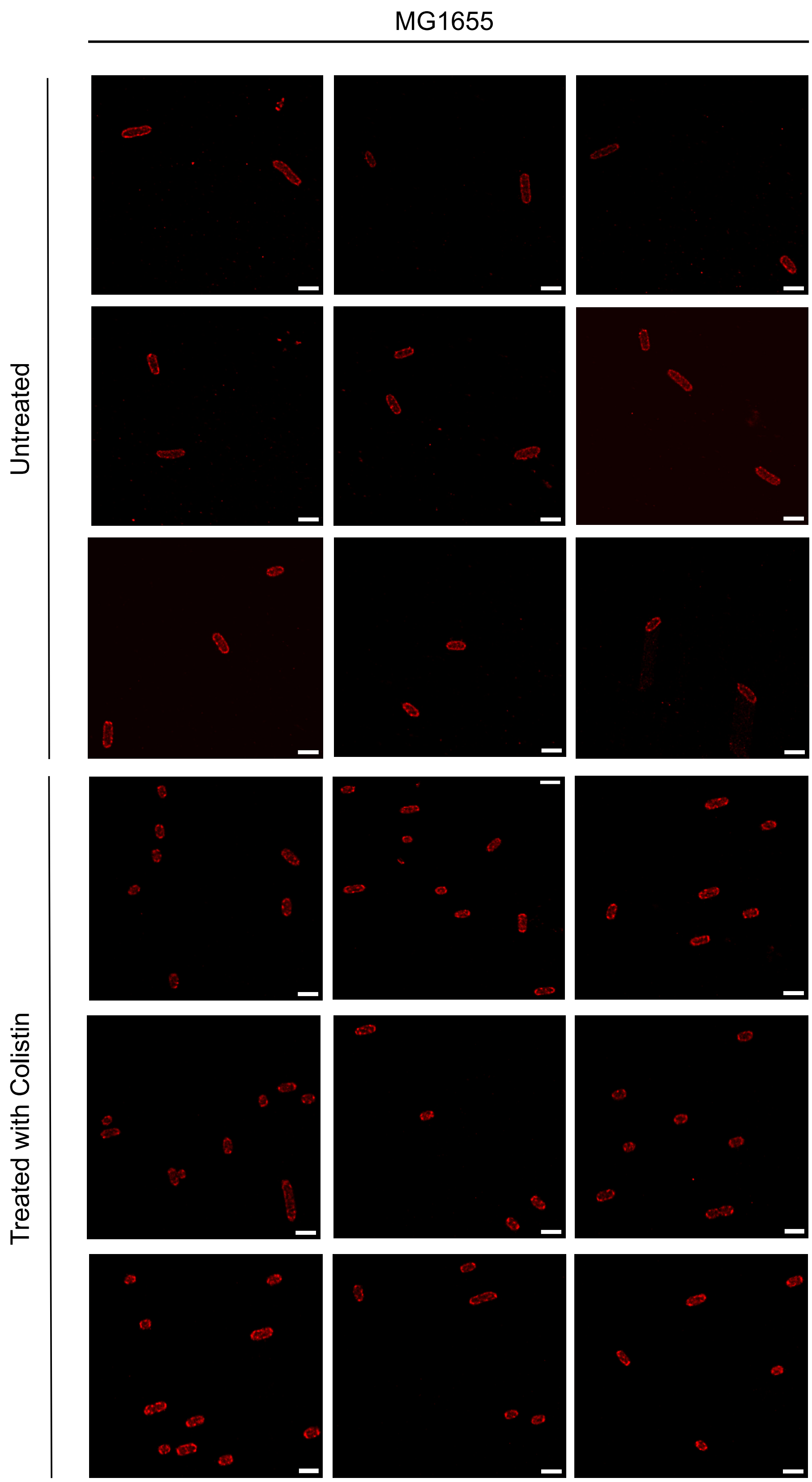


**Supplementary Figure 5. Visualisation of FM4-64-labelled membrane lipids in *E. coli* MG1655 under colistin-untreated and treated conditions with filtration**. The scale bars are 2 μm.

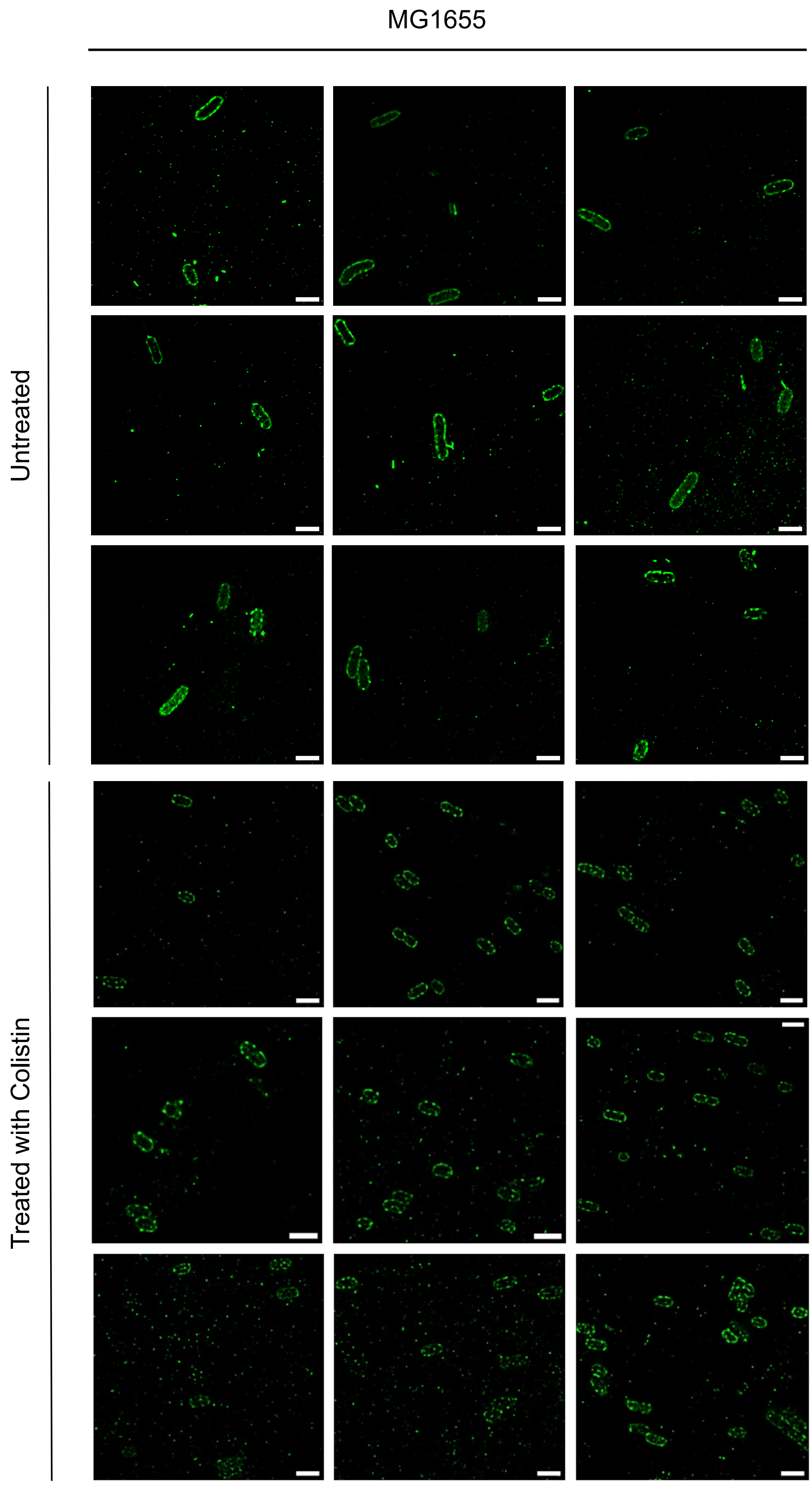


**Supplementary Figure 6. Visualisation of RB-PMB-bound LPS in *E. coli* MG1655 under colistin-untreated and treated conditions with filtration.** The scale bars are 2 μm.

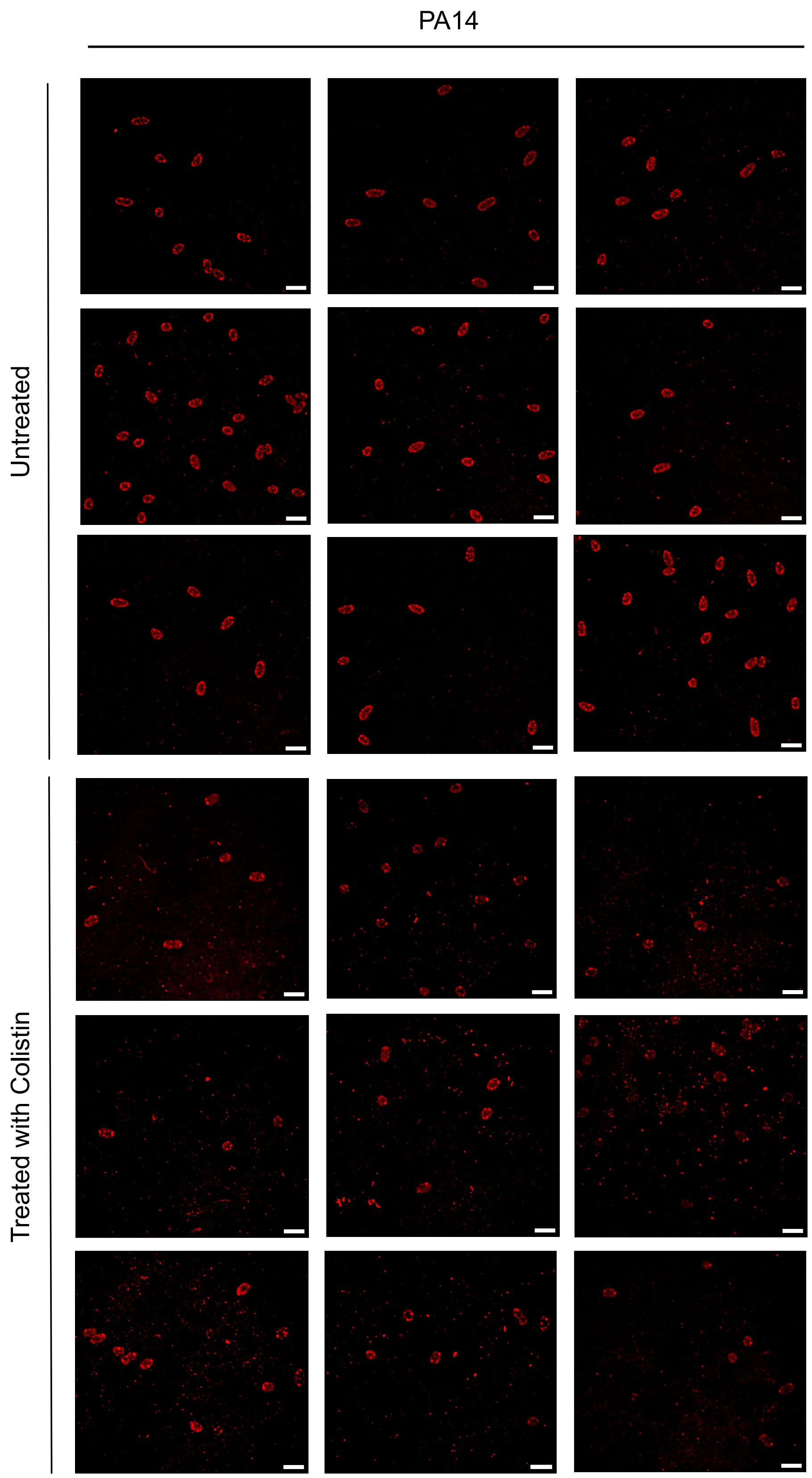


**Supplementary Figure 7. Visualisation of FM4-64-labelled membrane lipids in *P. aeruginosa* PA14 under colistin-untreated and treated conditions with filtration.** The scale bars are 2 μm.

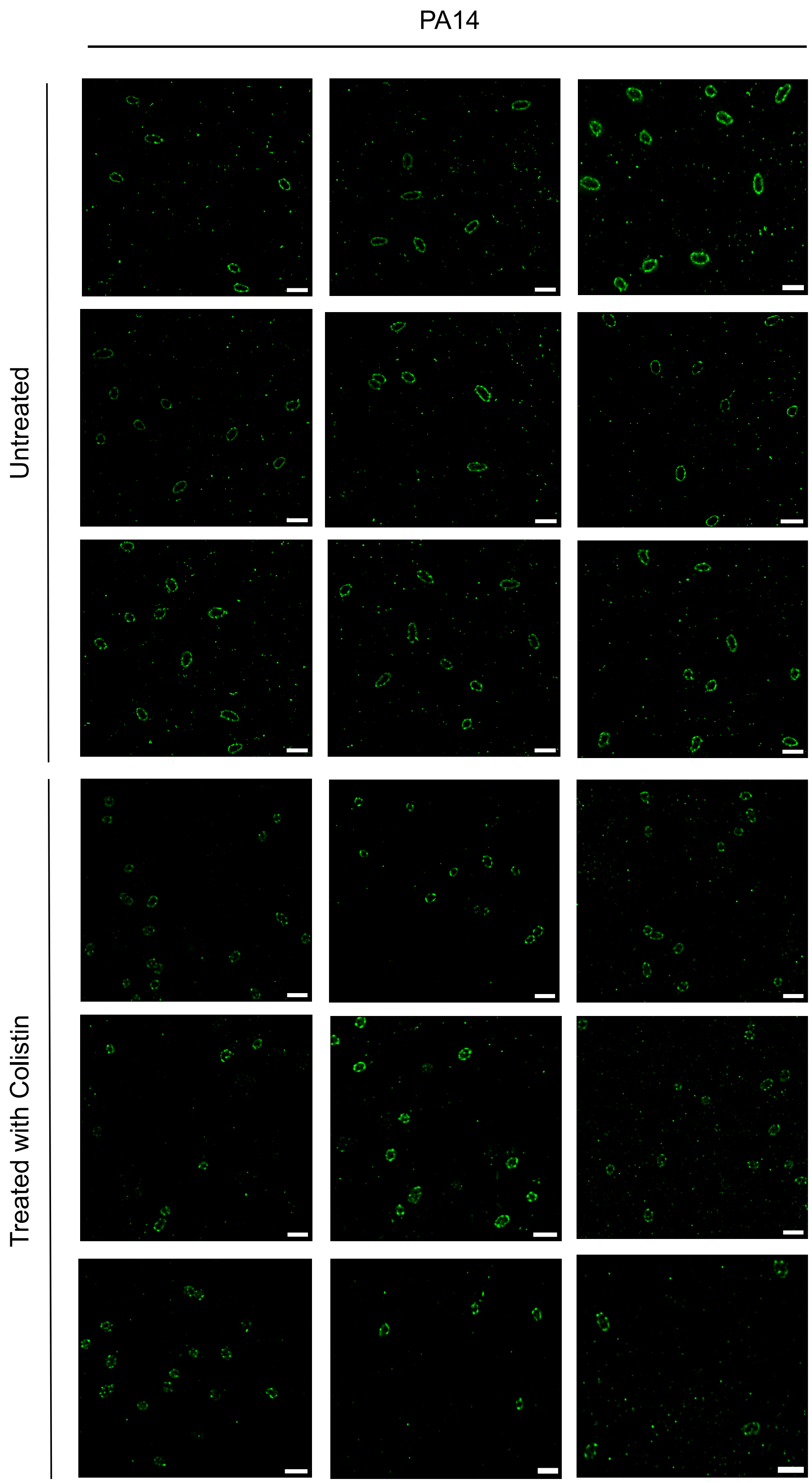


**Supplementary Figure 8. Visualisation of RB-PMB-bound LPS in *P. aeruginosa* PA14 under colistin-untreated and treated conditions with filtration.** The scale bars are 2 µm.

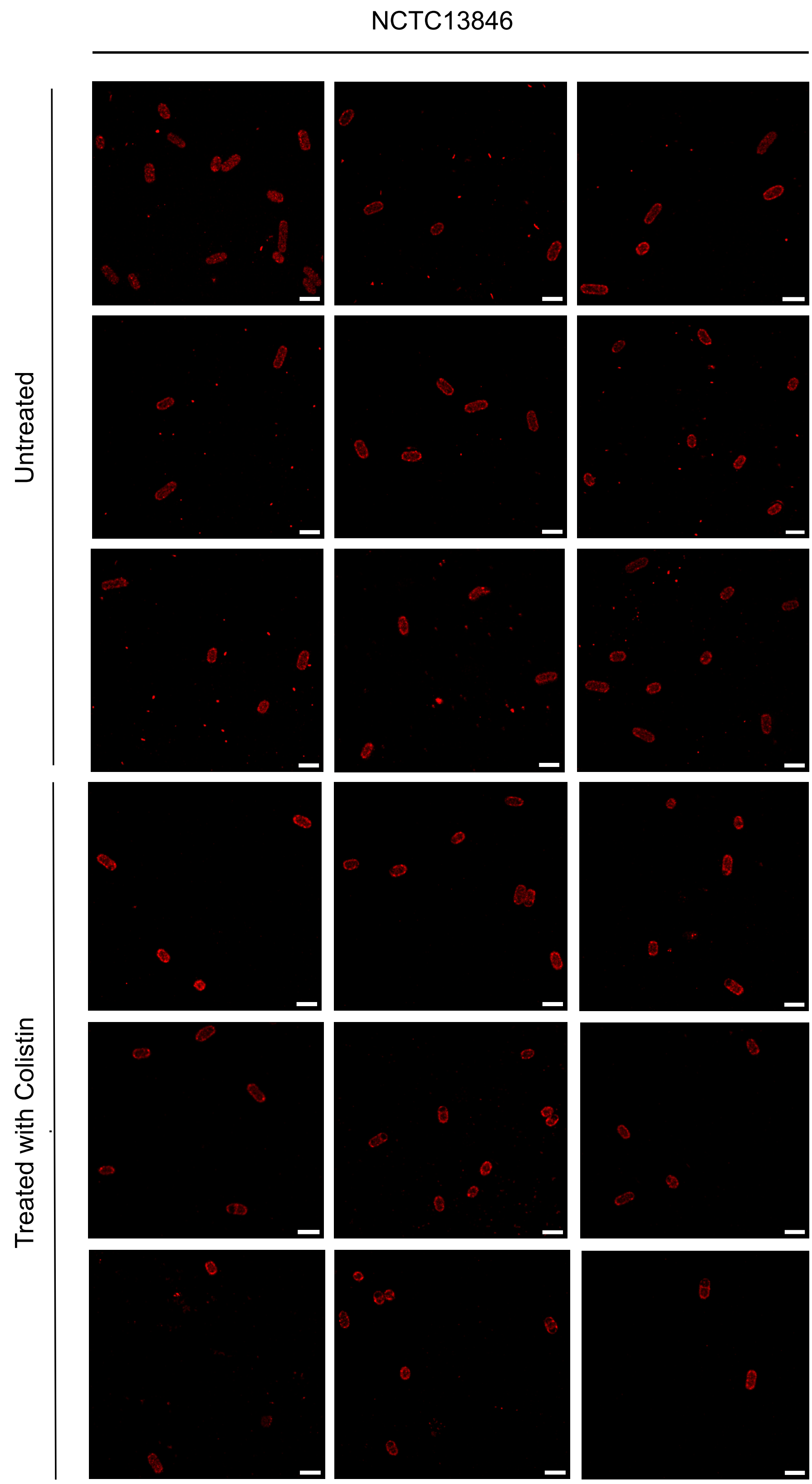


**Supplementary Figure 9. Visualisation of FM4-64-labelled membrane lipids in *E. coli* NCTC13846 under colistin-untreated and treated conditions with filtration.** The scale bars are 2 μm.

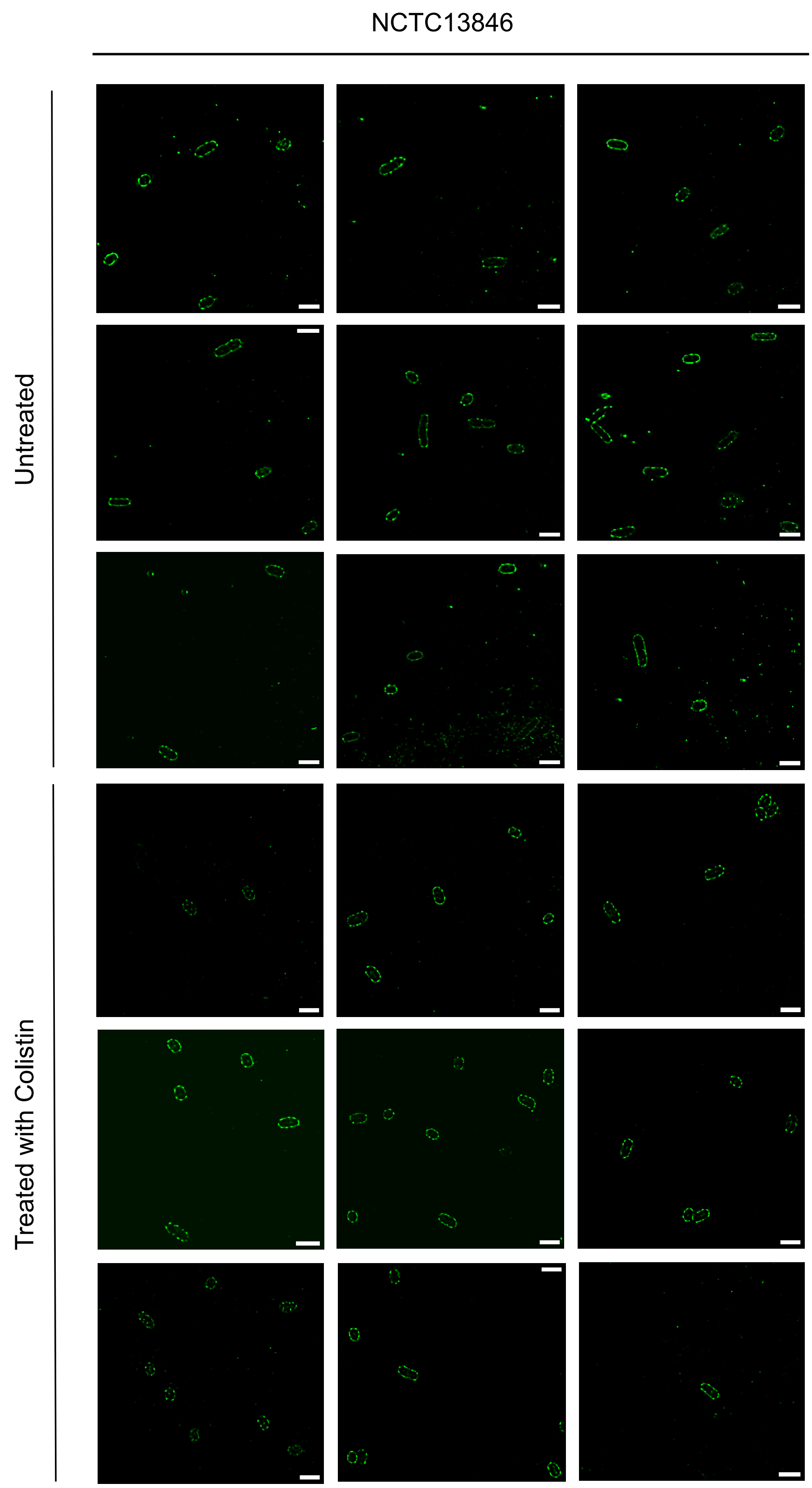


**Supplementary Figure 10. Visualisation of RB-PMB-bound LPS in *E. coli* NCTC13846 under colistin-untreated and treated conditions with filtration.** The scale bars are 2 μm.

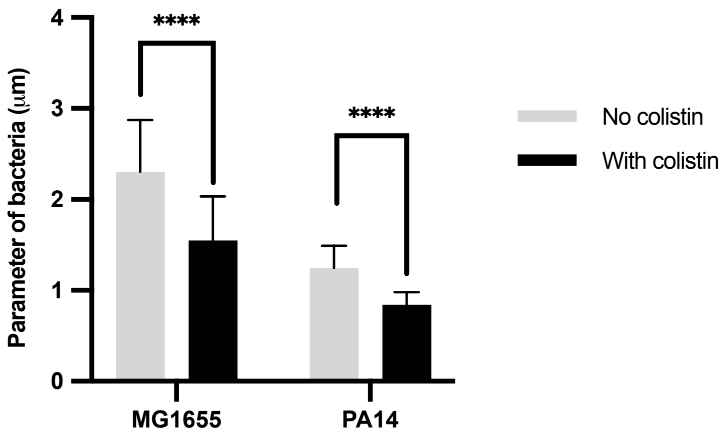


**Supplementary Figure 11. Comparison of cell size (perimeter) between susceptible and persistent cells**. Error bar indicates standard deviation of the mean (n = 20). Statistical significance calculated using unpaired t test. ****, $P \leq 0.0001$.

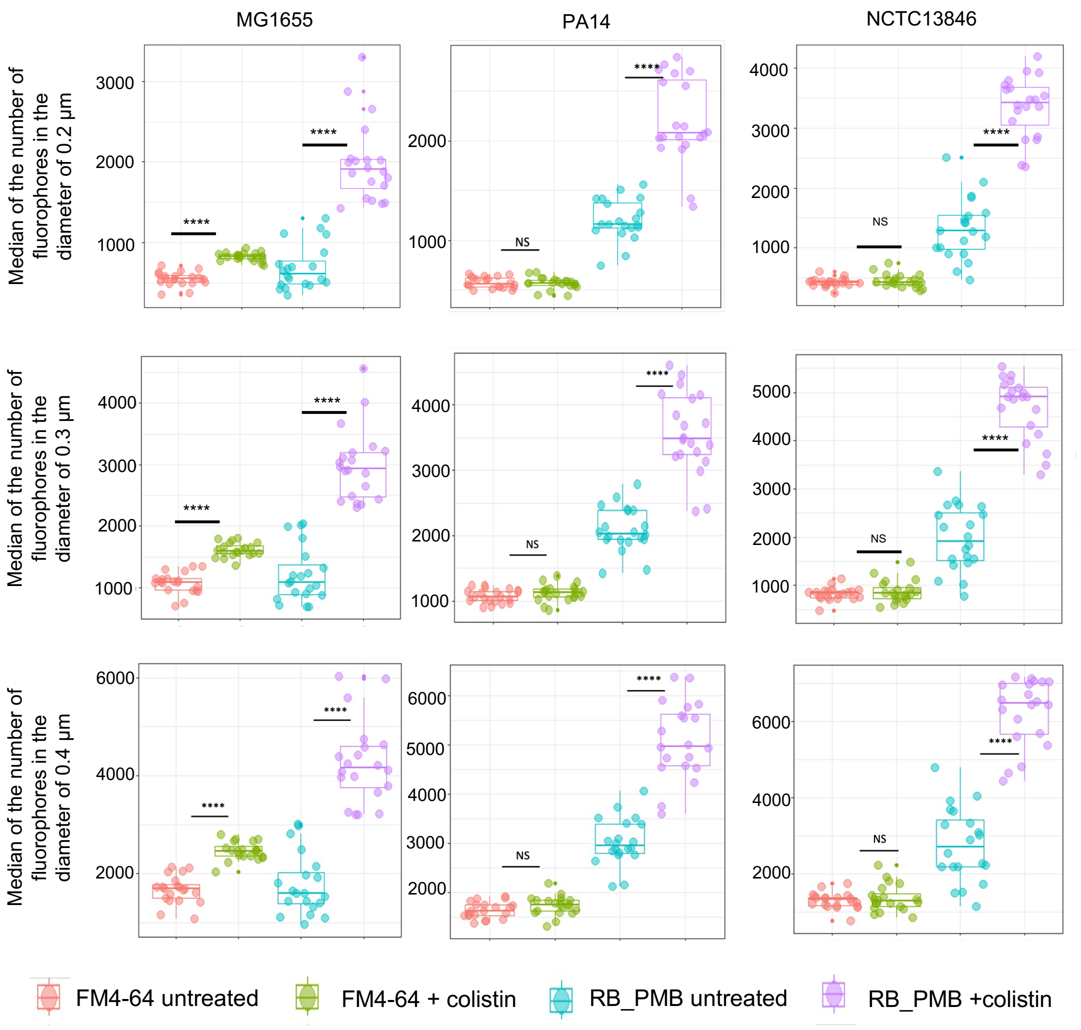


**Supplementary Figure 12. Fluorophore density within different ranges of diameter.** The plots present the median count of fluorophores detected within specified spatial ranges (0.2 µm, 0.3 µm, and 0.4 µm) in *E. coli* MG1655, *P. aeruginosa* PA14, and *E. coli* NCTC13846. Each point represents a single cell (n = 20 cells per condition). In the box plots, the horizontal line indicates the median; box edges represent the interquartile range (IQR); whiskers extend to 1.5 × IQR, and outliers are plotted as points. Statistical significance was evaluated using the Wilcoxon test with Bonferroni correction. NS (non-significant) P > 0.05); ****, P ≤ 0.0001.

**Supplementary Table 1. The number of bacteria cells exhibiting negative skewness levels across bacteria strains and conditions.** This table presents the count of bacterial cells with negative skewness in fluorescence intensity distribution, corresponding to different bacterial strains (*E. coli* NCTC13846, *E. coli* MG1655, and *P. aeruginosa* PA14) and experimental conditions (untreated versus colistin-treated). For each condition, there were 20 bacteria cells in total.

| Bacteria strains | FM4-64 | | Rhodamine B-labelled polymyxin B | |
|---|---|---|---|---|
| | Untreated | Colistin | Untreated | Colistin |
| NCTC13846 | 2 | 3 | 0 | 0 |
| MG1655 | 0 | 1 | 1 | 0 |
| PA14 | 0 | 2 | 0 | 0 |

**Supplementary Tables 2 to 4** present the exact measured values for variance, nearest-neighbour distances, and the total number of fluorophores within defined spatial ranges (0.1 µm to 0.4 µm) for both FM4-64-labelled membrane lipids and RB-PMB-bound LPS. The variance quantifies the heterogeneity of the fluorophore distribution along the bacterial envelope, while nearest-neighbour distances provide insights into the clustering of molecules. The fluorophore count reflects the molecular density across specific spatial scales. Comparisons between untreated and colistin-treated conditions are shown, along with the ratio of colistin to untreated values to indicate the magnitude of treatment-induced changes. These precise values capture the detailed responses of lipid and LPS distributions to colistin stress. Data are derived from 20 individual bacterial cells per condition.

**Supplementary Table 2. Precise quantification of lipid and LPS distribution and aggregation in *E. coli* MG1655 under untreated and colistin-treated conditions.**

| MG1655 | | FM4-64 | | | Rhodamine B labelled polymyxin B | | |
|---|---|---|---|---|---|---|---|
| | | Susceptible bacteria | Persisters | Persistent/Susceptible Ratio | Susceptible bacteria | Persisters | Persistent/Susceptible Ratio |
| Median of the average variance (n = 20) of fluorophores (units) | | 28.59 | 71.25 | $\sigma_p^2/\sigma_s^2$ = 2.49 | 132.38 | 2041.06 | $\sigma_p^2/\sigma_s^2$ =15.42 |
| Mean of the median (n = 20) of the nearest distance (μm) | | 0.012 | 0.010 | 0.83 | 0.010 | 0.005 | 0.50 |
| Mean of the median (n = 20) of the number of fluorophores in defined distance (units) | 0.1 μm | 162.65 | 233.30 | 1.43 | 236.98 | 827.40 | 3.49 |
| | 0.2 μm | 554.28 | 826.68 | 1.49 | 696.60 | 1988.00 | 2.85 |
| | 0.3 μm | 1072.88 | 1611.05 | 1.50 | 1203.55 | 2989.33 | 2.48 |
| | 0.4 μm | 1677.10 | 2466.75 | 1.47 | 1777.08 | 4266.63 | 2.40 |

**Supplementary Table 3. Precise quantification of lipid and LPS distribution and aggregation in *P. aeruginosa* PA14 under untreated and colistin-treated conditions.**

| PA14 | | FM4-64 | | | Rhodamine B labelled polymyxin B | | |
|---|---|---|---|---|---|---|---|
| | | Susceptible bacteria | Persisters | Persistent/Susceptible Ratio | Susceptible bacteria | Persisters | Persistent/Susceptible Ratio |
| Median of the average variance (n = 20) of fluorophores (units) | | 33.02 | 63.61 | $\sigma_p^2/\sigma_s^2$ =1.93 | 220.58 | 1589.05 | $\sigma_p^2/\sigma_s^2$ = 7.20 |
| Mean of the median (n = 20) of the nearest distance (µm) | | 0.011 | 0.012 | 1.09 | 0.008 | 0.005 | 0.63 |
| Mean of the median (n = 20) of the number of fluorophores in defined distance (units) | 0.1 µm | 173.20 | 154.90 | 0.89 | 394.35 | 812.03 | 2.06 |
| | 0.2 µm | 574.75 | 568.30 | 0.99 | 1201.65 | 2200.50 | 1.83 |
| | 0.3 µm | 1078.23 | 1129.40 | 1.05 | 2088.23 | 3571.08 | 1.71 |
| | 0.4 µm | 1633.58 | 1729.20 | 1.06 | 3024.60 | 5087.98 | 1.68 |

**Supplementary Table 4. Precise quantification of lipid and LPS distribution and aggregation in *E. coli* NCTC13846 under untreated and colistin-treated conditions.**

| NCTC13846 | | FM4-64 | | | Rhodamine B labelled polymyxin B | | |
|---|---|---|---|---|---|---|---|
| | | Untreated | Colistin | Colistin/Untreated Ratio | Untreated | Colistin | Colistin/untreated Ratio |
| Median of the average variance of fluorophores (n = 20) (units) | | 18.69 | 23.08 | $\sigma_c^2/\sigma_u^2$ = 1.23 | 910.97 | 12058.06 | $\sigma_c^2/\sigma_u^2$ = 13.24 |
| Mean of the median of the nearest distance (n = 20) (μm) | | 0.0139 | 0.0134 | 0.96 | 0.0065 | 0.0031 | 0.47 |
| Mean of the median of the number of fluorophores (n = 20) in defined distance (units) | 0.1 μm | 124.08 | 132.23 | 1.07 | 528.00 | 1759.88 | 3.33 |
| | 0.2 μm | 425.80 | 452.30 | 1.06 | 1312.48 | 3354.25 | 2.56 |
| | 0.3 μm | 823.95 | 875.05 | 1.06 | 1975.90 | 4678.80 | 2.37 |
| | 0.4 μm | 1290.23 | 1353.73 | 1.05 | 2757.18 | 6250.75 | 2.27 |
